# Reviewing the quality, health benefit and value for money of chemotherapy and targeted therapy for metastatic breast cancer

**DOI:** 10.1007/s10549-017-4374-6

**Published:** 2017-07-08

**Authors:** Xavier Ghislain Léon Victor Pouwels, Bram L. T. Ramaekers, Manuela A. Joore

**Affiliations:** Department of Clinical Epidemiology and Medical Technology Assessment (KEMTA), Care and Public Health Research Institute (CAPHRI) of the Faculty of Health, Medicine and Life Sciences of Maastricht University (FHML), Maastricht University Medical Centre, P.O. Box 5800, 6202 AZ Maastricht, The Netherlands

**Keywords:** Breast neoplasms, Neoplasm metastasis, Models, Economic, Cost-benefit analysis, Quality-adjusted life-years, Review

## Abstract

**Purpose:**

To provide an overview of model characteristics and outcomes of model-based economic evaluations concerning chemotherapy and targeted therapy (TT) for metastatic breast cancer (MBC); to assess the quality of the studies; to analyse the association between model characteristics and study quality and outcomes.

**Methods:**

PubMED and NHS EED were systematically searched. Inclusion criteria were as follows: English or Dutch language, model-based economic evaluation, chemotherapy or TT as intervention, population diagnosed with MBC, published between 2000 and 2014, reporting life years (LY) or quality-adjusted life-year (QALY) and an incremental cost-effectiveness ratio. General characteristics, model characteristics and outcomes of the studies were extracted. Quality of the studies was assessed through a checklist.

**Results:**

24 studies were included, considering 50 comparisons (20 concerning chemotherapy and 30 TT). Seven comparisons were represented in multiple studies. A health state-transition model including the following health states: stable/progression-free disease, progression and death was used in 18 studies. Studies fulfilled on average 14 out of the 26 items of the quality checklist, mostly due to a lack of transparency in reporting. Thirty-one per cent of the incremental net monetary benefit was positive. TT led to higher iQALY gained, and industry-sponsored studies reported more favourable cost-effectiveness outcomes.

**Conclusions:**

The development of a disease-specific reference model would improve the transparency and quality of model-based cost-effectiveness assessments for MBC treatments. Incremental health benefits increased over time, but were outweighed by the increased treatment costs. Consequently, increased health benefits led to lower value for money.

**Electronic supplementary material:**

The online version of this article (doi:10.1007/s10549-017-4374-6) contains supplementary material, which is available to authorized users.

## Introduction

Worldwide, breast cancer is the most incident and prevalent cancer among women (data from 2012) [1]. Due to the incurable character of metastatic breast cancer (MBC) and the intensive health care resource use associated with its management, MBC treatment incurs a high burden on health care budgets [2]. Policy makers therefore resort to economic evaluations to take coverage decisions concerning MBC treatments [3]. These economic evaluations are often based on decision-analytic models (or cost-effectiveness models) because different sources of evidence need to be synthesised and extrapolation of trial results is required to estimate the (lifetime) costs and the impact on survival and quality of life of MBC treatments. Health benefits obtained from MBC treatments are then weighted against their costs, which provide a measure of value for money used in MBC treatments.

Throughout the years, cost-effectiveness models have increasingly been used to support reimbursement decision for new (MBC) treatments and guidelines on good modelling practices have been developed [4, 5]. However, differences in model structure and assumptions, which might influence the cost-effectiveness outcomes [6], still exist between cost-effectiveness models for MBC treatments [7–10]. Study sponsorship and quality have also been reported to influence the results of cost-effectiveness assessments. Industry-sponsorship was associated with more beneficial cost-effectiveness outcomes for the treatments of interest, while higher study quality was associated with less favourable cost-effectiveness outcomes [11]. Previous research also found that the quality of the cost-effectiveness assessments concerning oncology treatments has not increased over time [12]. More specifically, a previous review concerning cost-effectiveness models for MBC treatments highlighted the need for high-quality studies [13].

Because model design influences cost-effectiveness results, researchers and the European network for health technology assessment (Eunethta) have argued for increased consistency between cost-effectiveness assessments [14–17]. Eunethta consequently encourages adherence to the HTA Core model^®^ [18] and researchers have argued for the development of disease-specific reference models; a unique model which would be used for all economic evaluations in a specific disease area [19, 20].

A previous review of cost-effectiveness assessments evaluating chemotherapy and TT for MBC treatment has focussed on identifying the most influential characteristics of the included economic evaluations on the cost-effectiveness outcomes [13]. However, this previous review did not only include model-based economic evaluations, did not provide an overview of model characteristics, did not assess the quality of the included studies through a standardised checklist and did not attempt to illustrate the influence of different model characteristics on study quality and outcomes. The current study consequently aims at (1) providing an overview of model characteristics and outcomes of model-based economic evaluations of chemotherapy and TT for MBC treatment, (2) assessing the quality of the included studies and (3) investigating the association between model characteristics and study quality and outcomes.

## Methods

### Literature search and study selection

PubMed and the National Health Services Economic Evaluation Database (NHS EED) were searched through September and October 2014 (22-10-2014). Existing reviews [13, 21–24] informed the PubMed search query which followed the PICO methodology (patient, intervention, comparator, outcome) (Online Resource, Appendix 1). The NHS EED search query was composed of the following terms: “Breast cancer” OR “Breast neoplasm”. Inclusion criteria were:

- The study population includes patients diagnosed with advanced or MBC.
- The study is a model-based economic evaluation.
- Chemotherapy or TT is included as a comparator.
- The study reports an incremental cost-effectiveness ratio (ICER) with life years (LYs) and/or quality-adjusted life years (QALYs) as measure of effect.
- The study has been published in English or Dutch as a journal article between January 2000 and October 2014.

XP performed abstract screening. During full text screening, XP reviewed all studies, while BR and MJ each reviewed half of the studies. Disagreements about inclusion were resolved through discussions among all authors. XP performed reference tracking in order to retrieve potentially relevant studies. Inclusion of studies without abstract was assessed during full-text screening.

### Extraction of general information, model characteristics and outcomes

XP retrieved general information on authors, country, year of publication, comparators, perspective and sponsorship of each study. Through a standardised extraction sheet, the authors retrieved the model characteristics: type of model (the health state-transition model category was composed of “Markov” state-transition models and partitioned survival models), health states, treatment effect modelling (constant or time-dependent), time horizon, extrapolation beyond trial time horizon, cycle time, adverse events (AEs) (AEs were considered as included when either costs or the effects on quality of life of AEs were incorporated in the model) and subgroup analyses included in the economic evaluations. This was performed in duplicates and discrepancies were discussed among all authors. XP also registered which treatment lines were under investigation in each study. When the treatment line was not clearly stated in the text, it was labelled as ‘unclear/mix’ because studies might investigate a treatment which is administrated in different treatment lines.

XP extracted information on model inputs: utilities, utility elicitations methods, the type of AEs included and the population (hormonal and human epidermal growth factor receptor 2 (HER-2)-statuses). The following study outcomes were extracted: total LY, QALY and costs for each comparator, incremental costs and effects (incremental LY (iLY) and/or incremental QALY (iQALY)) and ICERs. Total costs, incremental costs and ICERs were converted to the year 2013 by using the Consumer Price Index of each country [25–27]. Costs were adjusted to US$ 2013 and then to € 2013 by using the Purchase Power Parity [28]. ICERs were rounded to the nearest thousand (or hundred if smaller than 1000). The Net Monetary Benefit (NMB) of each comparator and the incremental NMB (iNMB) of each comparison at a willingness-to-pay threshold of €40,000 per QALY were calculated.

### Quality assessment

Quality assessment of the studies was performed based on a previously used checklist [23] which consisted of the CHEC checklist [29] and additional items suggested by Soto [30]. These additional items concern the type of model, the description of the model and the source of data used in the model. Quality indicators were scored as follows: yes/complete details given in text (1); no/no details given (0); not clearly stated within text, references given (N.C.) and not applicable (N.A.) [23]. Two authors assessed each study (XP and BR or MJ). Disagreements were resolved through discussions among all authors. The number of items rated as ‘yes/complete details given’ were summed up for each study in order to obtain an indication of study quality. The checklist contained 26 items.

### Association of model characteristics with study quality and outcomes

Graphic plots were used to investigate the association between study quality and study sponsorship, publication year, iQALY and iNMB. Study quality was represented in percentage of correctly described items (‘yes/complete details given in text’) from the quality checklist. Furthermore, the association between study outcomes (iNMB and iQALY) and publication year as well as time horizon was explored. A lifetime time horizon was defined as 20 years, as this approximates lifetime in this condition. Finally, the association between iQALY and iNMB was investigated.

## Results

### Literature search

**Fig. 1:**
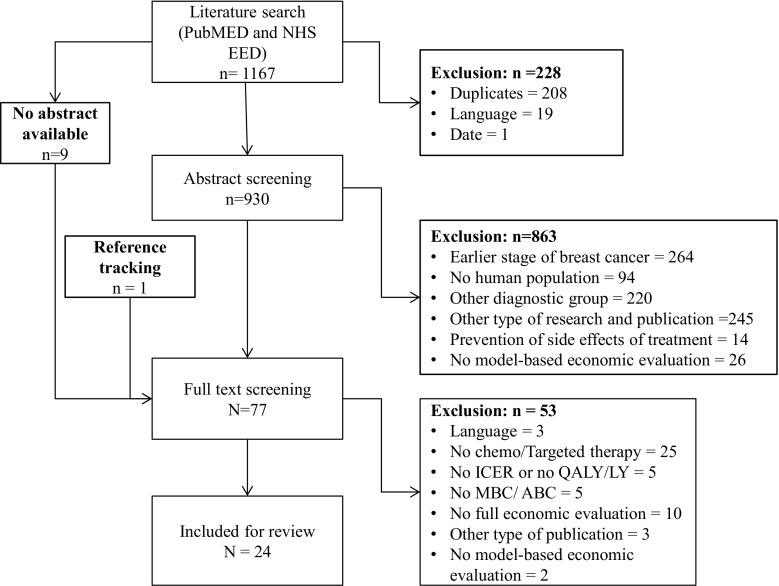
Consort diagram of the selection procedure. *ABC* advanced breast cancer, *chemo* chemotherapy, *ICER* incremental cost-effectiveness ratio, *LY Life years, MBC* metastatic breast cancer, *QALY* Quality-adjusted life years

The literature search provided 1167 records. From those, 208 were duplicates, 19 were excluded based on language restrictions, 1 was excluded based on its publication date and the abstracts of 9 studies were not available. This resulted in 930 records eligible for abstract screening; of those, 863 were excluded. Full-text screening was performed on 77 articles (67 studies identified through abstract screening, 9 studies without abstract and 1 potential relevant study identified through reference tracking [31]). Twenty-four studies [8, 9, 32–53] were included (Fig. 1).

### General information and models’ characteristics of the studies

Studies were performed in Europe (*N* = 14), North America (*N* = 9) and South America (*N* = 1). Funding by a pharmaceutical company was reported by 11 studies. Two studies used only LY as outcome for the cost-effectiveness assessment, 10 used only QALY and 12 used both LY and QALY. Three studies used a societal perspective, twenty a health care/payer perspective and one used both societal and health care perspectives. The populations in the studies differed with respect to hormonal status and HER-2 status. Studies investigated interventions in different treatment lines (Table 1). The 24 studies provided 50 comparisons of treatments: 20 concerned chemotherapy and 30 concerned TT. Seven specific treatment comparisons were represented in multiple studies, totaling 20 comparisons, six of them being the same comparisons presented from two different perspectives (health care and societal). The remaining comparisons were only reported in one of the included studies.

**Table 1:** General and model characteristics of the included studies

Study	Population HR and HER2 status	Country	Publication year	Treatment line (previous treatment)	Type of model	Health states	Perspective	Time horizon	Extrapolation	Cycle time	Cost categories
Alba et al. [8]^a^	N.S.	Spain	2013	Second line (Anthracycline or other N.S.)	HSTM	PFD; PD; death	Health care	5 years	Yes (Weibull)	3 weeks	Medication, administration, monitoring, general care(health state costs), terminal phase, AEs
Athanasakis [53]^a^	HER-2+	Greece	2012	First line	HSTM	PFD; PD; death	Health care	12 years	No	1 month	Medication, administration, supportive care, AEs
Benedict et al. [32]^a^	N.S.	UK	2009	Unclear/mix (Anthracycline)	HSTM	PFD; PD; death	Health care	10 years (lifetime)	Yes (three-parameter gamma)	3 weeks	Medication, administration, terminal phase, progression diagnosis, post-progression chemotherapy, BSC, AEs
Brown et al. [33]^a^	N.S.	UK	2001	Second line (Anthracycline)	HSTM	Response; PFD; PD; death	Health care	3 years	No	3 weeks	Medication, administration, hospitalisation, visits, monitoring, palliative medication, AEs
Dedes et al. [34]^c^	N.S.	Switzerland	2009	First line metastatic setting	HSTM	PFD; PD; death	Health care	Lifetime	No	1 month	Medication, concomitant medication during chemotherapy, monitoring, disease progression, AEs
Delea et al. [9]^a^	Post-menopausal, HR+ , HER-2+	Canada	2013	First line	HSTM	PFD; PD; death	Societal & Health care	10 years	Yes (Weibull)	1 day	Medication, administration, monitoring, pre- and post-progression, AEs, direct non-medical, indirect costs
Delea et al. [35].^a^	Post meno, HR+ , HER-2+	UK	2013	First line	HSTM	PFD; PD; death	Health care	10 years (lifetime)	Yes (Weibull)	1 day	Medication, administration, monitoring, pre and post-progression costs, AEs
Delea et al. [36].^a^	HER-2+	UK	2012	Unclear/mix (Trastuzumab)	HSTM	PFD; PD; death	Health care	5 years (lifetime)	Yes (Weibull)	1 day	Medication, administration, monitoring, pre and post-progression follow-up, AEs
Elkin et al. [37].^b^	HER-2+	US	2004	First line	HSTM	Response; PFD; PD; death	Societal	Lifetime	No	1 week	Medication, diagnosis, patient travel and time, visits, monitoring, progressive disease, AEs
Frias et al. [38].^a^	N.S.	Spain	2010	Unclear/mix (Anthracycline)	HSTM	PFD; PD; death	Health care	5 years	Yes(three-parameter gamma)	3 weeks	Medication, administration, progression diagnosis, best supportive care, end of life phase, AEs
Lazzaro et al. [39].^a^	N.S.	Italy	2013	Second line (N.R.)	HSTM	PFD; PD; death	Health care	5 years (lifetime)	Yes (Weibull)	3 weeks	Medication, administration, best supportive care, end of life phase, AEs
Le et al. [40].^c^	HER-2+	US	2009	Second line (Anthracycline, taxane, trastuzumab)	HSTM	Response; PFD; PD; death	Societal	Lifetime	No	1,5 month	Medication, monitoring, disease progression, AEs, patient time
Li et al. [41].^c^	N.S.	NL	2001	Second line (N.R.)	N.R.	Short term: Febrile Neutropenia, no Febrile Neutropenia, death; long term: response, non-response, PD, death	Health care	1 year	No	3 month	Medication, hospitalisation, follow-up
Lidgren et al. [42].^c^	HER-2+	Sweden	2008	First line	HSTM	PFD; PD; death	Societal	N.R.	No	1 month	Medication, visits, monitoring, diagnostics, AEs
Lopes et al. [43].^c^	N.S.	US	2013	Unclear/mix	Decision-tree and HSTM	Response; PFD; PD; death	Payer: Medicare	N.R.	No	21-day	Medication, visits, monitoring
Machado et al. [44].^a^	HER-2+	Brasil	2012	Unclear/mix (Trastuzumab)	HSTM	PFD; PD; death	Health care	5 years	Yes(Weibull)	1 month	Medication, visits, AEs
Matter-Walstra et al. [45].^b^	HER-2+	Switzerland	2010	Unclear/mix (Trastuzumab)	HSTM	PFD; PD; death	Health care	Lifetime	No	3 weeks	Drug, monitoring, progression, AEs
Montero et al. [46].^a^	N.S.	US	2012	First line (N.R.)	Decision-tree	Paclitaxel alone or bevacizumab + paclitaxel; further therapy and best supportive care; death	Payer	N.R.	No	N.A.	Medication, physician and administration fees, monitoring
Norum et al. [47].^c^	N.S.	Norway	2005	Unclear/mix (N.R.)	N.R.	N.A.	Third party payer	N.R.	No	N.A.	Medication, visits, monitoring, diagnostics, AEs
Reed et al. [48].^a^	N.S.	US	2009	Unclear/mix (Anthracycline)	Decision-tree	Response, PFD, PD, not determined	Health care	N.R.	No	N.A.	Medication, visits, hospitalisation, monitoring, subsequent treatment
Refaat et al. [49].^c^	HER-2-	US	2014	First line	HSTM	Metastatic breast cancer + Rx, bevacizumab and Rx complications, PD, death	Health care (and patient)	5 years	No	1 year	N.C.
Takeda et al. [50].^b^	N.S.	UK	2007	Second line (Anthracycline)	HSTM	Response; PFD; PD; death	Health care	Lifetime	Yes(Lognormal)	3 weeks	Medication, visits, administration, AEs
Verma et al. [51].^c^	N.S.	Canada	2003	Unclear/mix (Anthracycline)	N.C.	N.A.	Health care	N.C.	No	N.A.	Medication, visits, administration, AEs
Verma et al. [52].^a^	N.S.	US	2005	Unclear/mix (Anthracycline)	HSTM	PFD; PD; death	Payer (and patient); health care costs considered	2.9 years	No	3 weeks	Medication, administration, visits, AEs

*AEs* adverse events, *BSC* best supportive care, *HER-2* human epidermal growth factor receptor 2, *HR* hormone receptor, *HSTM* health state-transition model, *N.A.* not applicable, *N.C*. not clearly reported, *N.R.* not reported, *N.S.* not specified, *PD* progressive disease, *PFD* progression-free disease, *UK* United Kingdom, *US* United States

^a^Industry-sponsored

^b^Publicly financed

^c^Sponsor not reported

Most studies used a health state-transition model (*N* = 18). The remaining studies used a decision-tree (*N* = 2), a combination of decision-tree and health state-transition model (*N* = 1) or did not clearly report which type of model was used (*N* = 3). Most (18 out of 19) studies using a health state-transition model (either combined with a decision-tree or not) included at least the following three health states: stable/progression-free disease, progression and death. Six of these studies also incorporated a response health state. All studies included AEs, but the number and types of AE differed (Online Resource, Appendix 2). Two studies stated they included AEs but did not provide details on which (and how) AEs were incorporated in the model [51, 53]. Nine studies used a lifetime time horizon, nine studies used a fixed time horizon (varying between 1 and 12 years) and six studies did not clearly define or report their time horizon. Cycle time varied between one day to one and a half months (Table 1).

Extrapolation of trial data was described in nine studies. Six studies extrapolated survival data through a parametric survival model assuming a Weibull distribution, two assumed a gamma distribution and one assumed a lognormal distribution. All studies seemed to model treatment effectiveness by applying the hazard ratio of the alternative intervention to the survival function (Online Resource, Appendix 3). Lazzaro et al. was unclear about how treatment effectiveness was modelled [39]. None of the studies mentioned the use of a time-dependent treatment effect.

Health state utility values varied from 0.67 to 1.00, from 0.61 to 0.72 and from 0.26 to 0.68 for the response, stable/progression-free disease and progression health states, respectively. Different impacts on quality of life were associated with AEs (disutility range −0.03 to −0.25) (Online Resource, Appendix 4).

Three studies presented subgroup analyses: one was based on age categories [34], another on the number of chemotherapy lines received before the interventions under study [36] and the last focused on patients’ body mass and surface [45].

### Outcomes

Total LY and QALY ranged from 0.70 to 3.43 and from 0.29 to 2.64, respectively. Total costs ranged from €1983 to €86,174. The NMBs ranged from €−45,374 to €59,161 (*N* = 61) (Online Resource, Appendix 5). Incremental LY and QALY gained varied from 0.06 to 0.74 and from 0.05 to 0.60, respectively. In two comparisons, the intervention dominated the comparator [36, 44], and the intervention (extendedly) dominated the comparator in six comparisons [37, 42]. For the remaining comparisons, the ICERs varied between €200 and €164,000 per LY gained (*N* = 24) and between €300 and €625,000 per QALY gained (*N* = 40). The iNMBs ranged from €−78,574 to €15,890 (*N* = 48); 15 (31%) of these iNMBs were positive. Norum et al. [47] results are not included in this overview because it reported a range of ICERs per LY gained instead of the results of a base-case analysis (Table 2).

**Table 2:** Outcomes of the studies

Study	Intervention (regimen)		Comparator (regimen)		LYs gained^a^	QALYs gained^a^	Incremental costs^b^	ICER LY^b,c^	ICER QALY^b,c^	INMB
Benedict et al. [32].	Doc (3 wk)	–	Pac (3 week)	–	0.53	0.33	5670	11,000	17,000	7530
Brown et al. [33].	Doc (3 wk)	–	Pac (3 week)	–	N.R.	0.09	263	N.R.	3000	3337
Benedict et al. [32].	Doc (3 wk)	–	Pac (1 wk)	–	0.47	0.29	1901	4000	6000	9699
Frias et al. [38].	Doc (3 wk)	–	Pac (1 wk)	–	0.37	0.24	78	200	300	9522
Benedict et al. [32].t	Doc (3 wk)	–	Nab-pac (3 wk)	–	0.39	0.22	4521	12,000	21,000	4279
Brown et al. [33].	Doc (3 wk)	–	Vino (1 wk)	–	N.R.	0.25	5423	N.R.	21,000	4577
Li et al. [41].	Doc (3 wk)	–	M (6 wk)	V (3 wk)	0.06^d^	0.05	14,022	N.R.	279,000	−12,022
Verma et al. [51].	Cap (14,3 wk)	Doc (3 wk)	Doc (3 wk)	–	N.R.	N.R.	N.R.	3000	N.R.	N.C.
Verma et al. [52].	Cap (14, 3 wk)	Doc (3 wk)	Doc (3 wk)	–	0.22	0.15	2067	9000	14,000	3933
Lopes et al. [43].	Eribulin (N.S.)	–	TPC (N.S.)	–	0.21	0.12	20,141	97,000	169,000	−15,341
Lopes et al. [43].	Eribulin (N.S.)	–	Cap (N.S.)		0.21	0.12	15,762	76,000	132,000	−10,962
Lopes et al. [43].	Eribulin (N.S.)	–	Nab-pac (N.S.)		0.21	0.12	12,229	59,000	103,000	−7429
Lopes et al. [43].	Eribulin (N.S.)	–	Doxil (N.S.)		0.21	0.12	10,298	49,000	86,000	−5498
Lopes et al. [43].	Eribulin (N.S.)	–	Ixa (N.S.)		0.21	0.12	7239	35,000	61,000	−2439
Takeda et al. [50].	Gem (1,8, 3 wk)	Pac (3 wk)	Pac (3 wk)	–	0.32	0.16	13,743	43,000	85,000	−7343
Reed et al. [48].	Ixa (14,3 wk)	Cap (14,3 wk)	Cap (14,3 wk)	–	0.17^d^	0.09	26,326^g^	164,000	306,000	−22,726
Alba et al. [8].	Nab-Pac (3 wk)	–	Pac (3 wk)	–	0.27	0.16	3055	12,000	19,000	3345
Lazzaro et al. [39].	Nab-Pac (3x/week)	–	Pac (3x/week)	–	N.R.	0.17	2621	N.R.	16,000	4179
Li et al. [41].	Pac (3 wk)	–	M (6 wk)	V (3 wk)	0.06^d^	0.07	7142	N.R.	108,000	−4342
Li et al. [41].	Vino (1,8, 3 wk)	M (3 wk)	M (6 wk)	V (3 wk)	0.15^d^	0.14	3619	N.R.	25,000	1981
Dedes et al. [34].	Bev (1, and 15)	Pac (3 out of 4)	Pac (3 out of 4)	–	0.13^d^	0.21	40,098	N.R.	188,000	−31,698
Montero et al. [46].	Bev (N.S.)	Pac (N.S.)	Pac (N.S.)	–	N.R.	0.14	84,174	N.R.	625,000	−78,574
Refaat et al. [49].	Bev (N.S.)	Pac (N.S.)	Pac (N.S.)	–	N.R.	0.37	72,127	N.R.	195,000	−57,327
Delea et al. [36].	Lap (14,3 wk)	Cap (14,3 wk)	Cap (14,3 wk)	–	0.29	0.19	19,280	66,000	101,000	−11,680
Machado et al. [44].	Lap (14,3 wk)	Cap (14,3 wk)	Cap (14,3 wk)	–	0.29	0.19	31,241	66,000	165,000	−23,641
Le et al. [40].	Lap (14,3 wk)	Cap (14,3 wk)	Cap (14,3 wk)	–	0.16	0.12^d^	17,456	107,000	148,000	−12,656
Delea et al. [36].	Lap (14,3 wk)	Cap (14,3 wk)	Cap (14,3 wk)	Trast (3 wk)	0.19	0.31	−139	N.R.	Dominant	12,539
Machado et al. [44].	Lap (14,3 wk)	Cap (14,3 wk)	Cap (14,3 wk)	Trast (3 wk)	0.23	0.13	−10,690	Dominant	Dominant	15,890
Delea et al. [9].	Lap (N.S.)	Let (N.S.)	Let (N.S.)	–	0.54	0.44	42,854	79,000	97,000	−25,254^e^
Delea et al. [35].	Lap (N.S.)	Let (N.S.)	Let (N.S.)	–	0.58	0.47	44,219	N.R.	95,000	−25,419
Delea et al. [9].	Lap (N.S.)	Let (N.S.)	Let (N.S.)	–	0.54	0.44	39,572	73,000	90,000	−21,972^f^
Delea et al. [9].	Lap (N.S.)	Let (N.S.)	Ana (N.S.)	Trast (N.S.)	0.33	0.24	3711	11,000	16,000	5,889^e^
Delea et al. [35].	Lap (N.S.)	Let (N.S.)	Ana (N.S.)	Trast (N.S.)	0.74	0.25	7018	N.R.	28,000	2982
Delea et al. [9].	Lap (N.S.)	Let (N.S.)	Ana (N.S.)	Trast (N.S.)	0.33	0.24	1551	5000	7000	8,049^f^
Delea et al. [9].	Lap (N.S.)	Let (N.S.)	Ana (N.S.)	–	0.7	0.57	43,137	62,000	76,000	−20,337^e^
Delea et al. [35].	Lap (N.S.)	Let (N.S.)	Ana (N.S.)	–	0.35	0.6	45,821	N.R.	76,000	−21,821
Delea et al. [9].	Lap (N.S.)	Let (N.S.)	Ana (N.S.)	–	0.7	0.57	38,905	56,000	69,000	−16,105^f^
Matter-Walstra et al. [45].	Trast (3 wk)	Cap (14,3 wk)	Cap (14,3 wk)	–	0.58	0.35	34,013	58,819	98,424	−20,013
Athanasakis [53]	Trast (3 wk)	Doc (3 wk)	Doc (3 wk)		0.73	0.45	27,371	38,000	61,000	−9371
Norum et al. [47].	Trast (1 wk)	–	No Trast	–	0.3–0.7	N.R.	52,277	75,000–174,000	N.R.	N.C.
Elkin et al. [37].	HercepTest, trast for 3+		No test, chemo alone		0.09	0.06	8,121^d^	N.R.	Extendedly dominated	−5721
Elkin et al. [37].	HercepTest, confirm 2+ with FISH, chemo and trast for FISH+ and HT+		No test, chemo alone		0.11	0.08	11,018^d^	N.R.	Dominated	−7818
Elkin et al. [37].	HercepTest, trast and chemo for 2+ and 3+		No test, chemo alone		0.11	0.08	14,517^d^	N.R.	Dominated	−11,317
Elkin et al. [37].	No test: trast, and chemo		No test, chemo alone		0.12	0.09	36,790^d^	N.R.	Dominated	−33,190
Elkin et al. [37].	HercepTest, confirm 2+ and 3+ with FISH, chemo and trast for FISH+		No test, chemo alone		0.11	0.08	10,655	N.R.	128,000	−7455
Elkin et al. [37].	FISH, trast and chemo for positives		No test, chemo alone		0.12	0.09	11,718	N.R.	149,000	−8118
Lidgren et al. [42]	IHC test, trast and chemo for IHC 3+		Chemo alone		N.R.	0.13	6437	N.R.	Extendedly dominated	−1237
Lidgren et al. [42].	IHC test, trast and chemo for IHC 2+ and 3+		Chemo alone		N.R.	0.18	10,784	N.R.	Dominated	−3584
Lidgren et al. [42].	IHC test, FISH confirmation for 2+ and 3+ , trast and chemo for FISH+		Chemo alone		N.R.	0.18	8592	N.R.	49,000	−1392
Lidgren et al. [42]	FISH test, trast and chemo for FISH + patients		Chemo alone		N.R.	0.19	9445	N.R.	57,000	−1845

*N.R.* not reported, *N.S.* frequency of administration is not specified, *1 wk* weekly administration, *3wk* administration once each 3 weeks, *6 wk* administration once each 6 weeks, *3×/week* 3 times weekly, *1,8,3 wk* administration on days 1, 8, of 3 weeks cycle, *3 out of 4* administration on days 1,8,15 of 4 weeks cycle, *14, 3 wk* daily during 14 days every 3 weeks,*?* regimen not described, *1 and 15* administration on day 1 and 15 of 4 weeks cycle, *chemo*z chemotherapy, *trast* trastuzumab, *doc* docetaxel, *pac* paclitaxel, *nab-pac* albumin-bound paclitaxel, *vino* vinorelbine, *M* mitomycin, *V* vinblastine, *doxil* liposomal doxorubicin, *lap* lapatinib, *bev* bevacizumab, *cap* capecitabine, *let* letrozole, *gem* gemcitabine, *ixa* ixapebilone, *ana* anastrozole, *HT+* HercepTest positive

^a^As reported in the text

^b^In € 2013

^c^Rounded to nearest 1000th or 100th if smaller than 1000

^d^Calculated by the authors, based on the information from the study

^e^Health care perspective

^f^Societal perspective

^g^Undiscounted costs

### Quality assessment

Most of the studies clearly described their objective (*N* = 16; 67%), comparators (*N* = 21; 88%) and model assumptions (*N* = 22; 92%). A societal perspective was used in four studies (17%). It was unclear whether the model was appropriate for the decision problem in three studies (*N* = 3; 13%). In two of these studies, the model was not graphically represented and the possible transitions between health states were not clearly described [39, 52]. In the third study, all health states of the model were neither mentioned nor graphically represented (*N* = 1; 4%) [41]. This hampered the authors in assessing whether the model was appropriate for the decision problem. In two studies (8%), the model structure was not considered appropriate given the information provided. The first study did not consider costs incurred after disease progression and did not justify this choice [47]. The second study considered patients dying before treatment response assessment as ‘Undetermined response’. However, patients in the ‘Undetermined response’ of the provided decision tree could still be subject to toxicities or progression which seemed to influence the transition probabilities of patients surviving and having an ‘Undetermined response’ [48]. Twenty-one (88%) studies identified all relevant outcomes, and thirteen (54%) clearly stated the probabilities that outcomes would happen. Outcome measurement and valuation were not clearly described in 13 studies (54%). Thirteen (54%) studies clearly identified all important and relevant costs, eighteen (75%) correctly valued costs and seventeen (71%) appropriately discounted costs. Fifteen studies (63%) did not clearly describe how costs were measured. The authors were not able to assess the credibility and accuracy of the sources of all values in ten studies (42%) because these were not clearly reported. Deterministic and probabilistic sensitivity analyses were performed in 23 (96%) and 17 (71%) studies, respectively. Ethical and distributional issues were considered in one study (4%). None of the studies appropriately fulfilled all items of the quality assessment. Studies fulfilled on average 14 out of the 26 items of the checklist (range 7–20) (Online Resource, Appendix 6).

### Association of model characteristics with study quality and outcomes

**Fig. 2:**
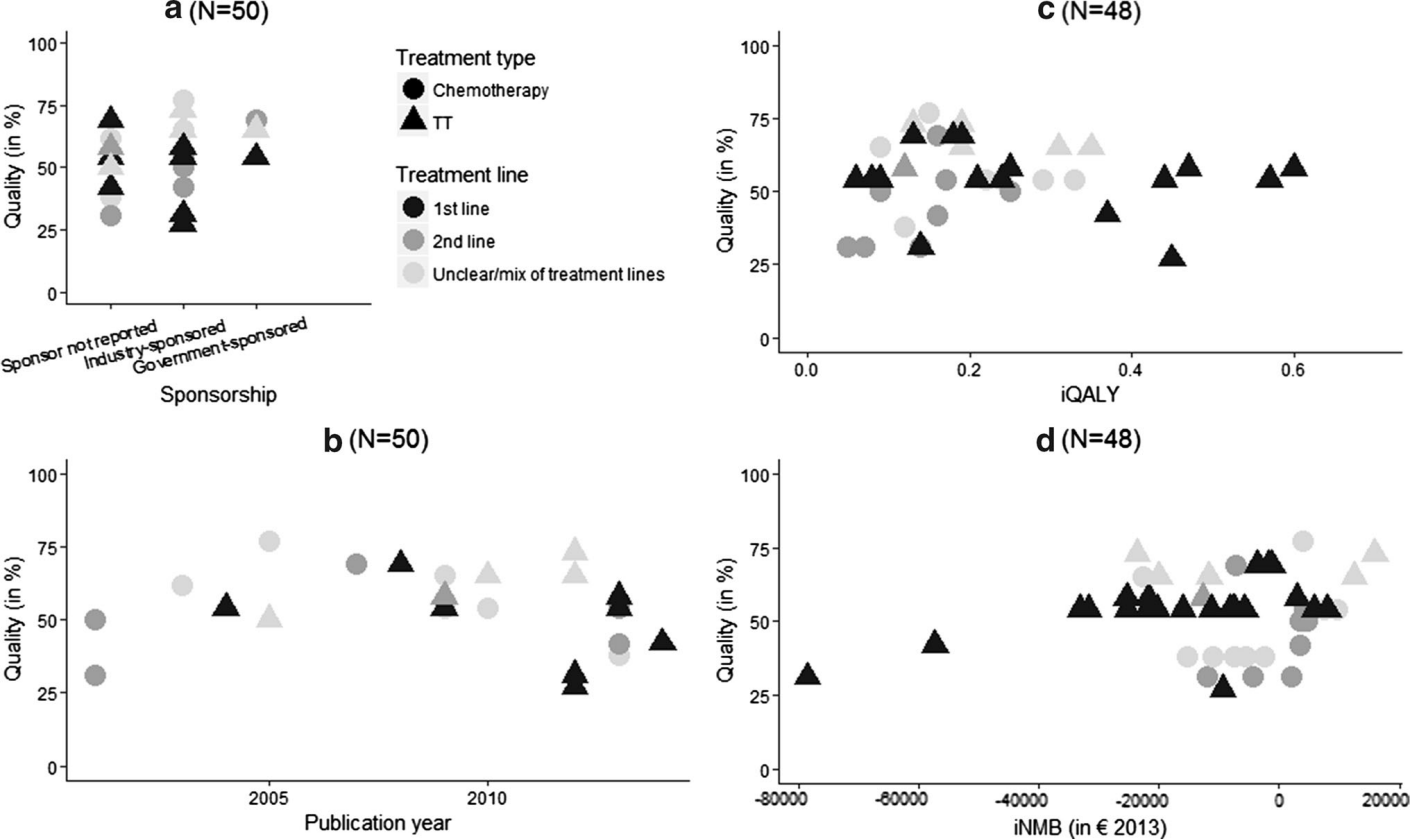
Association between study quality and study characteristics and between study quality and outcomes. **a** Association between study quality and study sponsorship; **b** association between study quality and publication year; **c** association between study quality and iQALY; **d** association between study quality and iNMB; *iQALY* incremental quality-adjusted life-year; *iNMB* incremental net monetary benefit

**Fig. 3:**
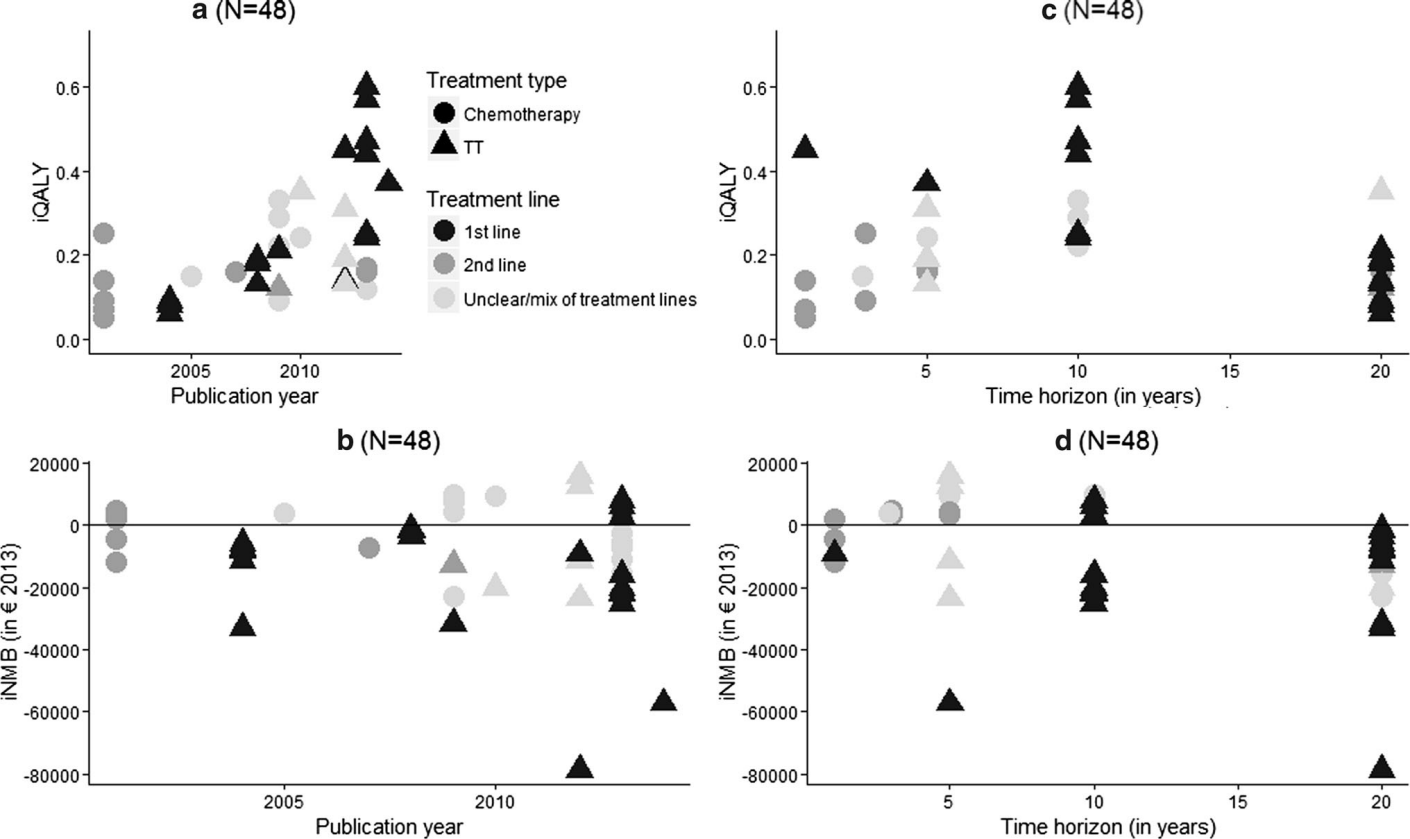
Association between model characteristics and study outcomes. **a** Association between iQALY and publication year; **b** association between study iNMB and publication year; **c** association between iQALY and time horizon; **d** association between iNMB and time horizon; *iQALY* incremental quality-adjusted life-year; *iNMB* incremental net monetary benefit

**Fig. 4:**
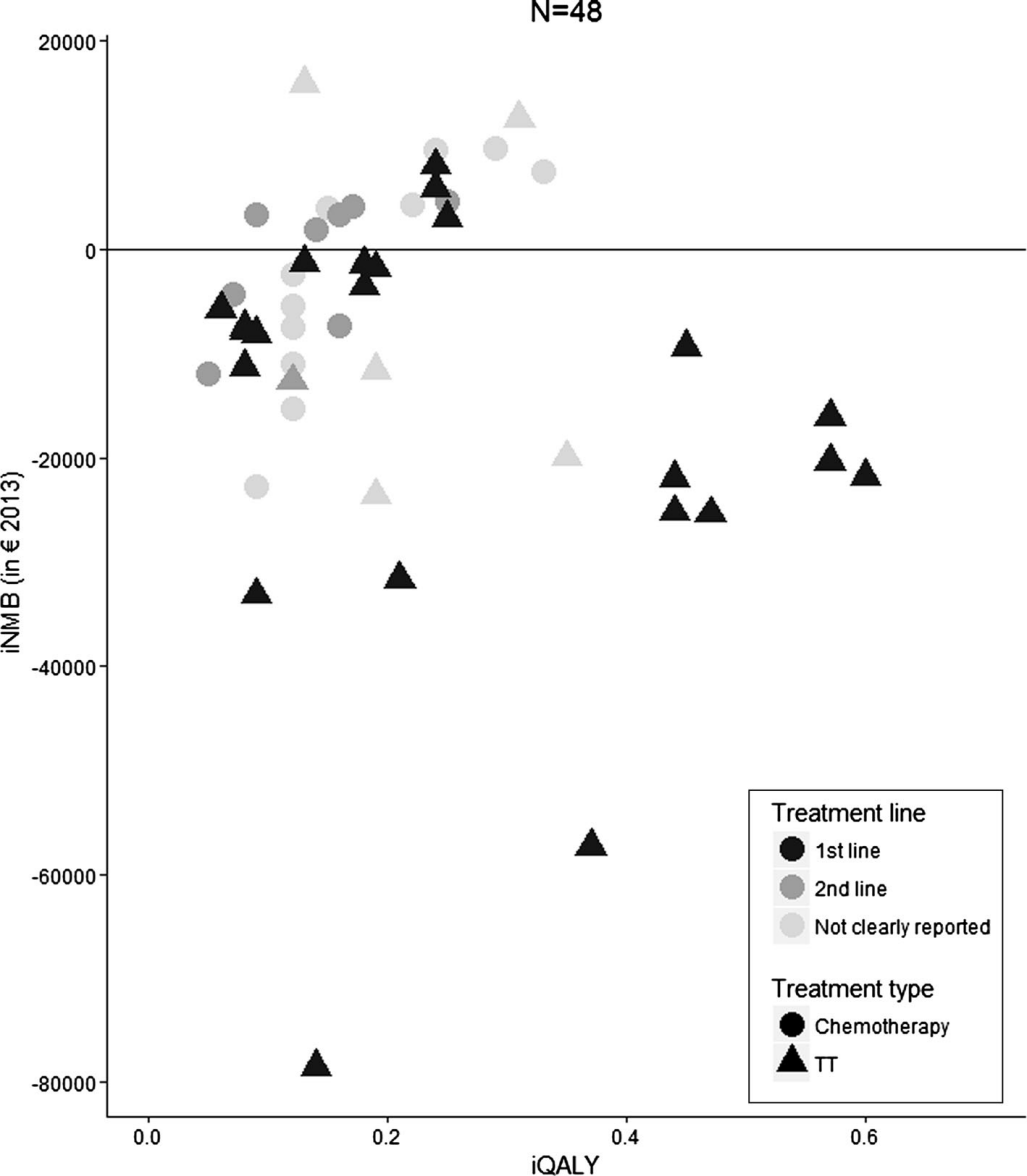
Association between iQALYs and iNMBs. *iQALY* incremental quality-adjusted life-year; *iNMB* incremental net monetary benefit

Study quality did not increase over time and did not seem to be associated with study sponsorship and outcomes (Fig. 2). Recently published studies more often investigated the cost-effectiveness of TT which led to higher iQALY (Fig. 3). Lifetime time horizon did not seem to lead to higher health benefits (Fig. 3). Fourteen out of the twenty-five (56%) industry-sponsored iNMBs were positive, while one of the 23 (4%) non-industry-sponsored iNMBs (sponsorship not reported or governmental sponsorship) was positive. Finally, increased iQALY seemed to be associated with a lower iNMB (Fig. 4).

## Discussion

The current literature review included 24 studies evaluating the cost-effectiveness of chemotherapy or TT for MBC treatment. Most studies (75%) used a health state-transition approach with three health states (stable/progression-free disease, progression and death) to model MBC, but differed with respect to time horizon, cycle times, AEs and utility values incorporated in the model. Quality of the studies was low and did not increase over time. iLY and iQALY gained ranged between 0.06 and 0.74, and 0.05 and 0.60, respectively. The iNMBs ranged from €−78,574 to €15,890 and 31% of the iNMBs were positive. TT led to higher iQALY gained. Industry-sponsored studies seemed to result in more favourable iNMB. Larger health benefits were not associated with higher value for money.

The results of the current literature study are subject to certain limitations. Firstly, the literature search was limited in time, publication type and language to make the number of included studies manageable and to retrieve up-to-date assessments potentially using state-of-the-art methodologies. Secondly, an adapted CHEC checklist, which was not specifically developed for model-based economic evaluations, was used for the quality assessment. However, this limitation is unlikely to influence our conclusions because more extensive checklists would also have identified the lack of transparency in reporting. Finally, the small number of studies investigating the same comparisons hampered comparisons of outcomes in relation to differences in model structure (e.g. number of health states) and model inputs. As a result, the consistency in outcomes between different comparisons could not be investigated.

The current study did not demonstrate an association between study quality and study outcomes or sponsorship. While this lack of association is reassuring, the absence of association between study quality and time, mainly due to transparency issues, is worrisome, especially because different guidelines concerning good modelling practices and reporting have been issued [4, 54]. Transparency is a hallmark of good modelling practices because it improves the ability to interpret results and it allows to examine the validity of the models and to reproduce model outcomes [4]. Reproducibility being an essential feature of medical research, (compulsory) disclosure of all model characteristics should be encouraged.

The development of a disease-specific reference model is another solution to resolve consistency, transparency and quality issues. Disease-specific reference models would avoid duplication of work across jurisdictions and potentially accelerate coverage decision-making for MBC treatments. It would furthermore decrease the methodological uncertainty associated with different modelling choices made during cost-effectiveness assessments of MBC treatments. Several authors have already attempted to develop such a reference model for MBC treatments. These models were however limited to a specific setting or patient population [19, 20].

Increased health benefits did not lead to higher value for money, which implies that treatment costs increased when health benefits became larger. This mechanism is typical of value-based pricing frameworks. However, one might expect that prices would be set in order to remain around the willingness-to-pay threshold in a value-based pricing setting. This was not the case in the current study, i.e. 31% of the iNMBs were positive. This might indicate that value-based pricing might be on its way in this field, but that lower prices are needed in order to meet the willingness-to-pay threshold. On the other hand, assessing the value of money for treatments in the metastatic setting only is misleading because using these treatments in the adjuvant setting [55] or using them more efficiently (e.g. because experience has been acquired in clinical practice) might provide better value for money. The potential value for money of these treatments over their entire life cycle may be underestimated by only assessing their value in the metastatic setting.

In conclusion, model inputs were highly variable and the quality of the included studies was low, mainly because of a lack of transparency in reporting. The development of a disease-specific reference model would increase the consistency and ensure a minimal quality of cost-effectiveness assessments for MBC treatments. Cost-effectiveness results were highly variable but, in general, MBC treatments did not provide good value for money. There was no association between study quality and study outcomes. Industry-sponsored studies resulted more often in beneficial value for money of treatments compared to non-industry-sponsored studies. TT led to larger health benefits. Incremental health benefits increased over time, but were outweighed by the increased treatment costs. Consequently, increased health benefits led to lower value for money.

## Electronic supplementary material

Below is the link to the electronic supplementary material.

Supplementary material 1 (PDF 1151 kb)
