# Supplementary material for: Reviewing the quality, health benefit and value for money of chemotherapy and targeted therapy for metastatic breast cancer
Source: Breast Cancer Res Treat. 2017 Jul 8;165(3):485–98. doi: 10.1007/s10549-017-4374-6 (PMC5602061; doi:10.1007/s10549-017-4374-6)
Supplement: Supplementary file 1 — Supplementary material 1 (PDF 1151 kb) [file 10549_2017_4374_MOESM1_ESM.pdf]

Reviewing the quality, health benefit, and value for money of chemotherapy and targeted therapy for metastatic breast cancer

Electronic supplementary material

Pouwels, X.G.L.V.[a]; Ramaekers, B.L.T.[a]; Joore, M.A.[a]

[a] Department of Clinical Epidemiology and Medical Technology Assessment (KEMTA), School for Public Health and Primary Care (CAPHRI) of the Faculty of Health, Medicine and Life Sciences of Maastricht University (FHML), Maastricht University Medical Centre

Journal: Breast Cancer Research and Treatment

Corresponding author: Xavier Pouwels (xavier.pouwels@mumc.nl)

*Appendix 1: Full search query PubMED and NHS EED***

P: Patient

(((Breast* OR mamma* OR ductal OR intraductal)
AND
(Tumor* OR tumour* OR cancer* OR carcinoma* OR recurrent OR advanced OR metasta* OR invasive OR adenocarcinoma* OR carcinoma OR sarcoma* OR oncolog* OR neoplas* OR malignan* OR lesion* OR growth* OR polyp* OR adenocystic OR "adenoid cystic" OR medullary OR mucinous OR colloid OR papillary OR metaplastic OR micropapillary))
OR
(Breast Neoplasms [MeSH Terms] OR Neoplasm Metastasis [MeSH Terms] OR mbc OR "invasive lobular cancer" OR ilc OR idc OR ibc OR "low-grade adenosquamous carcinoma"))

AND

Intervention and Comparator:

(Antineoplastic Agents [MeSH Terms]OR Radiotherapy [MeSH Terms] OR Molecular targeted therapy [MeSH Terms] OR Cytostatic Agents [MeSH Terms] OR Mastectomy, Segmental [MeSH Terms] OR Chemotherapy OR Chemotherapeutic OR Chemotherapeutic agent* OR "drug therapy" OR Radiotherap* and Chemotherapy OR "treatment combination" OR "radiation therapy" OR radiation OR immunotherapy OR chemoradiotherapy OR chemoradiotherap* OR chemo-radiotherap* OR "systemic therapy" OR "endocrine therapy" OR surgery OR surgical OR mastectomy OR hormone therapy)

AND

Outcome:

((Models, economic [MeSH Terms] OR Chain, markov [MeSH Terms] OR Models, Statistical [MeSH Terms] OR "decision analytic model" OR model* OR markov OR "discrete event simulation*" OR decision-tree*)

AND
(Economics, pharmaceutical [MeSH Terms] OR costs and cost analysis [MeSH Terms] OR cost* [title/abstract]) OR "Pharmacoeconomic*" [title/abstract] OR "Pharmaco economic*" [title/abstract]))

**NHS EED search was limited to the following terms: "Breast cancer" OR "Breast neoplasm", in order to retrieve all relevant studies, since this database only contains economic evaluation. Therefore, the economic evaluation-part of the PubMed search was not necessary.

Appendix 2: Quality assessment of the studies

**Studies:**	Alba et al. [1]	Athanasakis et al.[2]	Benedict et al. [3]	Brown et al.[4]	Dedes et al. [5]	Dele et al. [6]	Dele et al. [7]	Dele et al. [8]	Elkin et al. [9]	Firas et al. [10]	Lazzaro et al. [11]	Le et al. [12]	Li t al. [13]	Lidgren et al. [14]	Lopes et al. [15]	Machado et al. [16]	Matter-Walstra et al.[17]	Montero et al. [18]	Norum et al. [19]	Reed et al. [20]	Refaat et al. [21]	Takeda et al. [22]	Verma et al. [23]	Verma et al. [24]
Research question, perspective, and time horizon																								
Does a well-defined objective exist? Is it clear, explicit, and answerable?	1	0	1	1	0	0	1	1	0	1	1	0	1	0	0	1	0	1	1	1	1	1	1	1
Are competing alternatives described?	1	1	1	1	1	0	0	1	1	1	1	1	1	1	1	1	1	0	1	1	1	1	1	1
Is a societal perspective used?	0	0	0	0	0	1	0	0	1	0	0	1	0	1	0	0	0	0	0	0	0	0	0	0
Is it justified why the narrower perspective is valid?	0	0	0	0	0	N.A.	1	0	N.A.	0	0	N.A.	0	N.A.	0	1	0	0	0	0	0	0	1	1
Is a lifetime horizon taken into account?	0	0	1	0	1	0	1	1	1	0	1	1	0	1	0	0	1	0	N.C.	1	0	1	N.C.	0
Are reasons for another time horizon incorporated?	0	0	N.A.	0	N.A.	0	N.A.	N.A.	N.A.	0	N.A.	N.A.	0	N.A.	0	0	N.A.	0	0	N.A.	0	N.A.	0	1
Type and description of the model																								
Is the type of model used in the study stated clearly?	1	N.C.	1	1	1	1	1	1	1	1	1	1	0	1	1	1	1	1	0	1	1	1	0	1
Are details of	0	0	0	0	1	1	1	1	0	1	1	1	0	1	0	1	1	0	1	1	1	1	1	N.C.

**Studies:**	Alba et al. [1]	Athanasakis et al.[2]	Benedict et al. [3]	Brown et al.[4]	Dedes et al. [5]	Dele et al. [6]	Dele et al. [7]	Dele et al. [8]	Elkin et al. [9]	Firas et al. [10]	Lazzaro et al. [11]	Le et al. [12]	Li t al. [13]	Lidgren et al. [14]	Lopes et al. [15]	Machado et al. [16]	Matter-Walstra et al.[17]	Montero et al. [18]	Norum et al. [19]	Reed et al. [20]	Refaat et al. [21]	Takeda et al. [22]	Verma et al. [23]	Verma et al. [24]
the model given?																								
Is the design of the model appropriate and does it include the correct health states?	1	1	1	1	1	1	1	1	1	1	N.C.	1	N.C.	1	1	1	1	1	0	0	1	1	1	N.C.
Model data sources																								
Are the sources of all values credible and accurate?	N.C.	0	0	1	N.C.	1	0	1	1	N.C.	N.C.	N.C.	N.C.	N.C.	1	1	N.C.	1	1	N.C.	1	N.C.	1	1
Are assumptions incorporated into the model clearly stated?	N.C.	0	1	1	1	1	1	1	1	1	1	1	1	1	1	1	1	1	1	1	1	1	1	1
Outcomes and probabilities																								
Are all important and relevant outcomes for each alternative identified (LY or QALYs gained)?	1	1	1	1	1	1	1	1	1	1	1	0	1	1	0	1	1	1	1	1	0	1	1	1
Are the probabilities that outcomes happen clearly	0	0	0	N.C.	1	1	1	1	1	0	0	1	0	1	N.C.	1	1	0	0	1	0	1	1	1

**Studies:**	Alba et al. [1]	Athanasakis et al.[2]	Benedict et al. [3]	Brown et al.[4]	Dedes et al. [5]	Dele et al. [6]	Dele et al. [7]	Dele et al. [8]	Elkin et al. [9]	Firas et al. [10]	Lazzaro et al. [11]	Le et al. [12]	Li t al. [13]	Lidgren et al. [14]	Lopes et al. [15]	Machado et al. [16]	Matter-Walstra et al.[17]	Montero et al. [18]	Norum et al. [19]	Reed et al. [20]	Refaat et al. [21]	Takeda et al. [22]	Verma et al. [23]	Verma et al. [24]
stated?																								
Are all outcomes measured appropriately?	N.C.	N.C.	N.C.	0	N.C.	0	0	0	0	N.C.	N.C.	N.C.	N.C.	1	N.C.	1	N.C.	N.C.	N.C.	1	N.C.	1	1	1
Are outcomes valued appropriately?	N.C.	N.C.	N.C.	0	N.C.	0	0	0	0	N.C.	N.C.	N.C.	N.C.	N.C.	N.C.	1	N.C.	N.C.	N.C.	1	0	0	0	0
Costs																								
Are all important and relevant costs for each alternative identified?	N.C.	0	1	1	1	N.C.	N.C.	1	1	1	1	1	N.C.	1	0	1	1	0	1	0	N.C.	N.C.	N.C.	1
Are all costs measured appropriately in physical units?	N.C.	N.C.	N.C.	1	N.C.	N.C.	N.C.	N.C.	N.C.	N.C.	N.C.	N.C.	N.C.	1	1	N.C.	1	0	1	1	N.C.	N.C.	1	1
Are costs valued appropriately?	1	0	1	1	N.C.	1	1	1	1	1	1	1	N.C.	1	N.C.	1	1	0	1	1	N.C.	1	1	1
Are all future costs and outcomes discounted appropriately?	1	1	1	0	0	1	1	1	1	1	1	1	1	0	N.C.	1	1	0	1	1	0	1	N.A.	1
Incremental analysis and sensitivity analysis																								
Is an incremental analysis of cost	1	1	1	1	1	1	1	1	1	1	1	1	1	1	1	1	1	1	1	1	1	1	1	1

**Studies:**	Alba et al. [1]	Athanasakis et al.[2]	Benedict et al. [3]	Brown et al.[4]	Dedes et al. [5]	Dele et al. [6]	Dele et al. [7]	Dele et al. [8]	Elkin et al. [9]	Firas et al. [10]	Lazzaro et al. [11]	Le et al. [12]	Li t al. [13]	Lidgren et al. [14]	Lopes et al. [15]	Machado et al. [16]	Matter-Walstra et al.[17]	Montero et al. [18]	Norum et al. [19]	Reed et al. [20]	Refaat et al. [21]	Takeda et al. [22]	Verma et al. [23]	Verma et al. [24]
and outcomes of alternatives performed?																								
Is a one-way sensitivity analysis performed?	1	0	1	1	1	1	1	1	1	1	1	1	1	1	1	1	1	1	1	1	1	1	1	1
Is a probabilistic sensitivity analysis performed?	1	1	1	0	1	1	1	1	0	1	1	1	0	1	1	1	1	0	0	1	0	1	0	1
Discussion and conclusions																								
Do the conclusions follow from the data reported?	1	0	1	1	1	1	1	1	1	1	1	1	1	1	1	1	1	1	1	1	1	1	1	1
Does the study discuss the generalizability of the results to other settings and patients/clients groups?	0	1	0	0	0	0	0	0	0	0	0	0	0	1	0	0	0	0	0	0	0	1	1	1
Does the article indicate that there is no potential conflict of interest of study	0	0	0	0	1	0	0	0	N.C.	0	0	N.C.	N.C.	0	0	0	1	0	N.C.	0	1	1	N.C.	0

**Studies:**	Alba et al. [1]	Athanasakis et al.[2]	Benedict et al. [3]	Brown et al.[4]	Dedes et al. [5]	Dele et al. [6]	Dele et al. [7]	Dele et al. [8]	Elkin et al. [9]	Firas et al. [10]	Lazzaro et al. [11]	Le et al. [12]	Li t al. [13]	Lidgren et al. [14]	Lopes et al. [15]	Machado et al. [16]	Matter-Walstra et al.[17]	Montero et al. [18]	Norum et al. [19]	Reed et al. [20]	Refaat et al. [21]	Takeda et al. [22]	Verma et al. [23]	Verma et al. [24]
researcher(s) and funder(s)?																								
Are ethical and distributional issues discussed appropriately?	0	0	0	0	0	0	0	0	0	0	0	0	0	0	0	0	0	0	0	0	0	0	0	1
Legend: 1 = yes/complete details given in text; 0 = no/no details given; N.C. = not clearly stated within text, references given; N.A. = not applicable																								

Appendix 3: HRs sorted by regimens and studies

Studies	Intervention (administration schedule)		Comparator		HR PFS	HR OS	HR from progression to death
[3]	Doc (3wk)	-	Pac (3wk)	-	1.64	1.41	-
[4]	Doc (3wk)	-	Pac (3wk)	-	N.R.	N.R.	-
[13]	Doc (3wk)	-	M (6wk)	V (3wk)	N.R.	N.R.	-
[23]	Cap (14,3wk)	Doc (3wk)	Doc (3wk)	-	0.652	0.777	-
[24]	Cap (14,3wk)	Doc (3wk)	Doc (3wk)	-	N.R.	0.775	-
[15]	Eribulin (N.S.)	-	TPC (N.S.)	-	0.87	0.81	-
[22]	Gem (1,8, 3wk)	Pac (3wk)	Pac (3wk)	-	0.65	0.78	-
[20]	Ixa (14,3wk)	Cap (14,3wk)	Cap (14,3wk)	-	N.R.	N.R.	-
[1]	Nab-Pac (3wk)	-	Pac (3wk)	-	0.721	0.734	-
[3]	Nab-Pac (3wk)		Pac (3wk)		1.33	1.11	-
[11]	Nab-Pac (3x/week)	-	Pac (3x/week)	-	0.73-0.75[a]	0.73[a]	-
[13]	Pac (3wk)	-	M (6wk)	V (3wk)	N.R.	N.R.	-
[3]	Pac (1wk)		Pac (3wk)		1.09	1.04	-
[10]	Pac (1wk)	-	Doc (3wk)	-	1.64	1.41	-
[13]	Vino (1,8, 3wk)	M (3wk)	M (6wk)	V (3wk)	N.R.	N.R.	-
[5]	Bev (1, and 15)	Pac (3 out of 4)	Pac (3 out of 4)	-	0.6[b]	0.88[b]	-
[18]	Bev (N.S.)	Pac (N.S.)	Pac (N.S.)	-	N.R.	N.R.	-
[21]	Bev (N.S.)	Pac (N.S.)	Pac (N.S.)	-	N.R.	N.R.	-
[16]	Lap (14,3wk)	Cap (14,3wk)	Cap (14,3wk)	-	0.55	-	-
[16]	Cap (14,3wk)		Lap (14,3wk)	Cap (14,3wk)	-	1.33	-
[12]	Lap (14,3wk)	Cap (14,3wk)	Cap (14,3wk)	-	N.A.	N.A.	-
[8]	Lap (14,3wk)	Cap (14,3wk)	Cap (14,3wk)	-	0.55	-	-
[8]	Cap(14,3wk)		Lap (14,3wk)	Cap (14,3wk)	-	1.333	-
[6]	Lap (N.S.)	Let (N.S.)	Let (N.S.)	-	0.65	0.77	-
[7]	Lap (N.S.)	Let (N.S.)	Let (N.S.)	-	0.65	0.77	-
[6]	Trast	Ana	Let (N.S.)	-	0.73	0.90	-
[7]	Trast	Ana	Let (N.S.)	-	0.73	0.90	-
[2]	Trast	Doc	Doc	-	N.R.	N.R.	-
[6]	Ana		Let (N.S.)	-	1.22	1.08	-
[7]	Ana		Let (N.S.)	-	1.22	1.08	-
[8]	Trast (3wk)	Cap (14,3wk)	Cap (14,3wk)	-	0.685	0.763	-
[16]	Trast (3wk)	Cap (14,3wk)	Cap (14,3wk)		0.69	0.94	-
[17]	Trast (3wk)	Cap (14,3wk)	Cap	-	0.69	N.A.	1.035

Studies	Intervention (administration schedule)		Comparator		HR PFS	HR OS	HR from progression to death
			(14,3wk)				
[19]	Trast (1wk)	-	No Trast	-	N.A.	N.A.	-
[9]	HercepTest, trast and chemo for 3+		No test, chemo alone		N.R.	N.R.	-
[9]	HercepTest, confirm 2+ with FISH, chemo and trast for FISH + and HT+		No test, chemo alone		N.R.	N.R.	-
[9]	HercepTest, trast and chemo for 2+ and 3+		No test, chemo alone		N.R.	N.R.	-
[9]	No test: trast, and chemo		No test, chemo alone		N.R.	N.R.	-
[9]	HercepTest, confirm 2+ and 3+ with FISH , chemo and trast for FISH+		No test, chemo alone		N.R.	N.R.	-
[9]	FISH, trast and chemo for positives		No test, chemo alone		N.R.	N.R.	-
[14]	IHC test, trast and chemo for IHC 3+		Chemo alone		N.A.	N.A.	-
[14]	IHC test, trast and chemo for IHC 2+ and 3+		Chemo alone		N.A.	N.A.	-
[14]	IHC test, FISH confirmation for 2+ and 3+, trast and chemo for FISH+		Chemo alone		N.A.	N.A.	-
[14]	FISH test, trast and chemo for FISH+ patients		Chemo alone		N.A.	N.A.	-

[a] reported in introduction
[b] assuming it is based on ref 4 of the publication (Miller et al., E2100 trial [25])
Legend: ana= anastrozole; bev = bevacizumab; cap = capecitabine; chemo = chemotherapy; trast= trastuzumab; doc = docetaxel; doxil = liposomal doxorubicin; FISH = fluorescent in situ hybridization; gem = gemcintabine; HR = hazard ratio; HT+ = HercepTest positive; IHC= immunohistochemistry; ixa = ixapebilone; lap = lapatinib; let= letrozole; M = mitomicyn; nab-pac = albumin-bound paclitaxel; N.R.=not reported; N.S. = administration schedule not specified; pac= paclitaxel; PD = progressed disease; PFS = progression-free survival; N.A. = not applicable; V = vinblastine; vino = vinorelbine; 1wk= weekly administration; 3wk = administration once each 3 weeks; 6wk = administration once each 6 weeks; 3x/week= 3times weekly; 1,8,3wk = administration on days 1,8,of 3weeks cycle; 3 out of 4 = administration on days 1,8,15 of 4weeks cycle; 14,3wk = daily during 14days every 3weeks; 1 and 15 = administration on day 1 and 15 of 4 weeks cycle.

Appendix 4: AEs included in the studies (only studies reporting at least one AE are presented)

AEs/Study	[1]	[2][a]	[3]	[4]	[5]	[6]	[7][a]	[8]	[9][b]	[10][c]	[11]	[12]	[13]	[14]	[15]	[16]	[17]	[18]	[19]	[20]	[21]	[22]	[23][a]	[24][a]
**Neutropenia**	1			1							1									1!		1		1
**Febrile neutropenia**	1		1	1						1	1		1		1			1				1		
**Infection**	1		1	1	1					1	1													
**Anaemia**	1									1	1											1		
**Diarrhoea**	1		1			1		1		1	1	1			1	1		1				1		
**Nausea**	1		1			1		1		1	1		1											
**Vomiting &emesis**	1		1			1		1		1	1		1		1	1		1				1		
**Nervous system(neurosensitive, neuromotor) & neuropathy**	1		1	1						1	1				1			1				1		
**Stomatitis**	1		1							1	1													
**Pain & muscoloskeletal pain**	1		1			1				1	1													
**Asthenia**	1		1			1				1	1													
**Peripheral edema or oedema**	1		1	1						1														
**Myalgia**	1										1													
**Skin disorder (& rash)**			1	1		1		1		1														
**Nail disorder**			1	1		1				1														
**Cerebrovascular ischeamia**					1																1			
**Hypertension**					1	1															1			
**Hand foot syndrome**								1							1									
**Fatigue**						1		1							1			1				1		
**Left ventricular disfunction**								1																
**Alopecia**						1									1			1						
**Anorexia**						1																		
**Chills**						1																		

AEs/Study	[1]	[2][a]	[3]	[4]	[5]	[6]	[7][a]	[8]	[9][b]	[10][c]	[11]	[12]	[13]	[14]	[15]	[16]	[17]	[18]	[19]	[20]	[21]	[22]	[23][a]	[24][a]
**Constipation**						1																		
**Cough**						1																		
**Dyspnoea**						1																1		
**Epistaxis**						1																		
**Headache**						1															1			
**Hot flush**						1																		
**Nasopharyngitis**						1																		
**Pyrexia**						1																		
**Thrombocytopenia**																						1		
**Cardiac events (and congestive heart failure)**									1			1		1			1		1					
**Fatal infusion related allergic reaction**									1!															
**Proteinuria**																					1			
**Leucopenia**																						1		
**Elevated liver enzymes**																						1		
**Neutropenic fever/sepsis**																								1
**Total number of Aes reported**	**13**	**0**[a]	**12**	**7**	**3**	**20**	**0**	**7**	**1**	**13**	**12**	**2**	**3**	**1**	**7**	**2**	**1**	**6**	**1**	**0**[a]	**4**	**11**	**0**[a]	**2**

[a] no description of Aes included; in [24] neutropenia just appears in the results
[b] inclusion of cardiac events implied in the text, not clear if really taken into account
[c] anaemia only described in text, not in table with Aes

Appendix 5: Total cost, QALY, LY and NMB for each comparator

Studies	Publication year	Treatment	Total costs[a]	QALY gained	LY gained	NMB[a]
[1]	2013	Nab-pac (3wk)	17,104	0.80	1.44	14,896
[1]	2013	Pac (3wk)	14,049	0.64	1.17	11,551
[1]	2013	Pac (1wk)	17,844	0.80	1.44	14,156
[2]	2012	Doc (3wk)	9,129	1.54	2.70	52,551
[2]	2012	Trast+doc (3wk)	36,505	1.99	3.43	43,175
[3]	2009	Nab-pac (3wk)	19,910	0.96	1.62	18,490
[3]	2009	Pac (3 wk)	18,761	0.85	1.48	15,239
[3]	2009	Pac (1wk)	22,529	0.89	1.54	13,071
[3]	2009	Doc (3 wk)	24,431	1.18	2.01	22,769
[4]	2001	Pac (3 wk)	11,683	0.65	N.R.	14,257
[4]	2001	Doc (3 wk)	11,945	0.73	N.R.	17,443
[5]	2009	Pac (3 out of 4)	28,480	0.69	2.10	-880
[5]	2009	Pac (3 out of 4)+ bev (1 and 15)	68,578	0.90	2.23	-32,578
[6][b]	2013	Lap+let (N.S.)	69,650	2.30	3.27	22,430
[6][b]	2013	Let (N.S.)	26,796	1.86	2.72	47,684
[6][b]	2013	Trast+ana (N.S.)	65,939	2.07	2.94	16,701
[6][b]	2013	Ana (N.S.)	26,513	1.73	2.57	42,807
[6][c]	2013	Lap+let (N.S.)	79,632	2.30	3.27	12,448
[6][c]	2013	Let (N.S.)	40,060	1.86	2.72	34,420
[6][c]	2013	Trast+ana (N.S.)	78,081	2.07	2.94	4,559
[6][c]	2013	Ana (N.S.)	40,727	1.73	2.57	28,633
[7]	2013	Lap+let (N.S.)	77,160	2.39	3.40	18,400
[7]	2013	Let (N.S.)	32,942	1.92	2.82	43,978
[7]	2013	Trast+ana (N.S.)	70,142	2.14	2.66	15,338
[7]	2013	Ana (N.S.)	31,341	1.79	3.05	40,179
[8]	2012	Lap+cap (14,3wk)	37,460	0.93	1.65	-380
[8]	2012	Cap (14,3wk)	18,180	0.74	1.36	11,300
[8]	2012	Trast (3wk)+cap (14,3wk)	37,600	0.90	1.63	-1,760
[9]	2004	HercepTest, trast and chemo for 3+	44,428	1.28	2.34	6,772
[9]	2004	HercepTest, confirm 2+ with FISH, chemo and trast for FISH + and HT+	52,549	1.34	2.42	1,051
[9]	2004	HercepTest, trast and chemo for 2+ and 3+	55,084	1.36	2.44	-684
[9]	2004	No test: trast, and chemo	55,447	1.36	2.44	-1,047
[9]	2004	HercepTest, confirm 2+ and 3+ with FISH , chemo and trast for FISH+	58,945	1.36	2.44	-4,545
[9]	2004	FISH, trast and chemo for positives	56,146	1.37	2.45	-1,346
[9]	2004	No test, trast, and chemo	81,218	1.37	2.45	-26,418
[10]	2010	Pac (1wk)	21,853	0.84	1.46	11,747
[10]	2010	Doc (3wk)	21,930	1.08	1.83	21,270
[11]	2013	Nab-pac (3x weekly)	15,229	0.81	N.R.	16,971
[11]	2013	Pac (3x weekly)	12,609	0.64	N.R.	12,991
[12]	2009	Lap+cap (14,3wk)	59,133	N.R.	1.45	-

Studies	Publication year	Treatment	Total costs[a]	QALY gained	LY gained	NMB[a]
[12]	2009	Cap (14,3wk)	41,677	N.R.	1.29	-
[13]	2001	Pac (3wk)	11,507	0.35	0.76	2,493
[13]	2001	Doc (3wk)	18,421	0.34	0.76	-4,821
[13]	2001	Vino (1,8, 3wk) +M (3wk)	8,016	0.43	0.85	9,184
[13]	2001	M (6wk) +V (3wk)	4,397	0.29	0.70	7,203
[14]	2008	No test, chemo alone	33,502	1.28	N.R.	17,698
[14]	2008	IHC test, trast and chemo for IHC 3+	39,939	1.41	N.R.	16,381
[14]	2008	IHC test, trast and chemo for IHC 2+ and 3+	44,286	1.46	N.R.	13,954
[14]	2008	IHC test, FISH confirmation for 2+ and 3+, trast and chemo for FISH+	42,094	1.46	N.R.	16,146
[14]	2008	FISH test, trast and chemo for FISH+ patients	42,947	1.47	N.R.	15,893
[15]	2013	Eribulin (N.S.)	N.R.	N.R.	N.R.	-
[15]	2013	TPC (N.S.)	N.R.	N.R.	N.R.	-
[15]	2013	Cap (N.S.)	N.R.	N.R.	N.R.	-
[15]	2013	Nap-pac (N.S.)	N.R.	N.R.	N.R.	-
[15]	2013	Doxil (N.S.)	N.R.	N.R.	N.R.	-
[15]	2013	Ixa (N.S.)	N.R.	N.R.	N.R.	-
[16]	2012	Lap+cap (14,3wk)	55,251	0.96	1.70	-16,931
[16]	2012	Cap (14,3wk)	23,877	0.77	1.41	6,883
[16]	2012	Trast (3wk)+cap (14,3wk)	65,941	0.83	1.47	-32,861
[17]	2010	Cap (14,3wk)	23,239	1.17	2.06	23,561
[17]	2010	Trast (3wk)+cap (14,3wk)	57,253	1.51	2.64	3,147
[18]	2012	Pac (N.S.)	1,983	0.86	N.R.	32,217
[18]	2012	Pac+bev (N.S.)	86,174	1.02	N.R.	-45,374
[19]	2005	Trast (1wk)	52,277	N.R.	0.3-0.7	-
[20]	2009	Cap (14,3wk)	25,559	0.54	0.84	-4,159
[20]	2009	Ixa+cap (14,3wk)	51,885	0.62	1.01	-26,965
[21]	2014	Bev+chemo	N.R.	N.R.	N.R.	-
[21]	2014	Taxane based chemotherapy	N.R.	N.R.	N.R.	-
[22]	2007	Pac (3wk)	23,968	0.83	1.69	9,232
[22]	2007	Gem (1,8, 3wk) + pac (3wk)	37,711	1.00	2.01	2,289
[23]	2003	Doc (3wk)	10,059	N.R.	N.R.	-
[23]	2003	Cap (14,3wk) + doc (3wk)	10,706	N.R.	N.R.	-
[24]	2005	Doc (3wk)	23,258	0.66	1.16	3,142
[24]	2005	Cap (14,3wk) + doc (3wk)	25,325	0.81	1.38	7,075

[a] in € 2013
[b] health care perspective
[c] societal perspective
Legend: ana= anastrozole; bev = bevacizumab; cap = capecitabine; chemo = chemotherapy; trast= trastuzumab; doc = docetaxel; doxil = liposomal doxorubicin; FISH = fluorescent in situ hybridization; gem = gemcintabine; HR = hazard ratio; HT+ = HercepTest positive; IHC= immunohistochemistry; ixa = ixapebilone; lap = lapatinib; let= letrozole; M = mitomicyn; nab-pac = albumin-bound paclitaxel; N.R.=Not reported; N.S. = administration schedule not specified; pac= paclitaxel; PD = progressed disease; PFS = progression-free survival; V = vinblastine; vino = vinorelbine; 1wk= weekly administration; 3wk = administration once each 3 weeks; 6wk = administration once each 6 weeks; 3x/week= 3times weekly; 1,8,3wk = administration on days 1,8,of 3weeks cycle; 3 out of 4 = administration on days 1,8,15 of

Studies	Publication year	Treatment	Total costs[a]	QALY gained	LY gained	NMB[a]
4weeks cycle; 14,3wk = daily during 14days every 3weeks; 1 and 15 = administration on day 1 and 15 of 4 weeks cycle.						

Appendix 6: Details about elicitation method of utilities and population in which utilities were elicitated

Studies/Characteristics	Population where it was elicited	Instrument used for elicitation
[1]	N.R.	N.R.
[2]	N.R.	N.R.
[3]	N.R.	N.R.
[4]	Oncology nurses and nurses	SG
[5]	Canadian population	TTO
[6]	MBC patients, general pop (for the vignette based study)	FACT-G & FACT-B, vignette based study
[7]	MBC patients, general pop (for the vignette based study)	FACT-G & FACT-B, vignette based study
[8]	MBC patient (for pre-progression)	EQ-5D, utility decrement based on literature
[9]	US oncology nurses	N.R.
[10]	N.R.	N.R.
[11]	N.R.	N.R. (pooled analysis of MBC utility weights)
[12]	N.R.	N.R.
[13]	N.R.	N.R.
[14]	Swedish breast cancer patients	EQ-5D
[15]	N.R.	N.R.
[16]	MBC	EQ-5D (pre-progression), decrements
[17]	N.R.	N.R.
[18]	N.R.	N.R.
[19]	N.A.	N.A.
[20]	Patients	Health Utilities Index Mark 3 (HUI)
[21]	N.R.	N.R.
[22]	Oncology nurses anc clinical specialist	SG (study which is used is a pooled estimate from three studies using this methodology)
[23]	N.A.	N.A.
[24]	Nurses	SG (average of 3 studies)
Legend: EQ-5D = EuroQol - 5 dimension; FACT-B = Functional Assessment of Cancer Therapy - Breast; FACT-G = Functional Assessment of Cancer Therapy - General; MBC= metastatic breast cancer; N.A. = not applicable; N.R. = not reported; SG = standard gamble; TTO = time trade off		

Appendix 7: Utilities used in each study

Studies	Health states				Utility decrements for AEs
	Response	Pre-progression	Progression	Terminal disease	
[1]	0.810	0.650	0.450		0.11-0.21[a]
[2]		0.740	0.440		
[3]	0.790	0.720	0.450		0.06-0.07*
[4]	0.840	0.620	0.330	0.130	b
[5]		0.610	0.260		
[6]		0.860	0.620		0.03-0.15[a]
[7]		0.860	0.620		N.R.
[8]		0.694	0.472		
[9]	0.840	0.700	0.490		
[10]	0.790	0.720	0.450		0.06-0.07[g]
[11]	0.810	0.650	0.450		0.11-0.21[c]
[12]	0.840	0.700	0.500		
[13]	N.R.	N.R.	N.R.		
[14]	N.R.	0.685[d]	N.R.		
[15]	0.790	0.715[e]	0.443	0.190	0.103-0.155[a]
[16]		0.694	0.472		
[17]		0.700	0.500		
[18]	0.790	0.715	0.443		0.103-0.155[a]
[19]	N.A.	N.A.	N.A.		N.A.
[20]	0.670	0.650	0.610		
[20]	0.770	0.620	0.680		
[21]	1.000		0.500		0.250[b]
[22]	0.810 (0.670[f])	0.650 (0.540[f])	0.450 (0.450[f])		
[23]	N.A.	N.A.	N.A.		
[24]	N.A.	0.720	0.480		
**Minimum**	0.670	0.610	0.260	0.130	0.03-0.250
**Maximum**	1.000	0.860	0.680	0.190	

[a] each AE has a different utility decrement
[b] each AE is valued separately. Each has its own utility value assigned
[c] AE and state-specific
[d] called 'Metastatic breast cancer' in paper
[e] called 'Baseline' in paper
[f] with toxicities
[g] treatment-specific
Legend: N.A. = not applicable; N.R. = not reported

References

1. Alba E, Ciruelos E, Lopez R, Lopez-Vega JM, Lluch A, Martin M et al. Cost-utility analysis of nanoparticle albumin-bound paclitaxel versus paclitaxel in monotherapy in pretreated metastatic breast cancer in Spain. Expert review of pharmacoeconomics & outcomes research. 2013;13(3):381-91. doi:10.1586/erp.13.18.
2. Athanasakis K, J K. A cost-effectiveness analysis of trastuzumab plus docetaxel vs. docetaxel alone for the treatment of HER2-positive metastatic breast cancer in the Greek healthcare setting. Forum of Clinical Oncology. 2012;2012(3):4.
3. Benedict A, Cameron DA, Corson H, Jones SE. An economic evaluation of docetaxel and paclitaxel regimens in metastatic breast cancer in the UK. PharmacoEconomics. 2009;27(10):847-59. doi:10.2165/10899510-000000000-00000.
4. Brown RE, Hutton J, Burrell A. Cost effectiveness of treatment options in advanced breast cancer in the UK. PharmacoEconomics. 2001;19(11):1091-102.
5. Dedes KJ, Matter-Walstra K, Schwenkglenks M, Pestalozzi BC, Fink D, Brauchli P et al. Bevacizumab in combination with paclitaxel for HER-2 negative metastatic breast cancer: an economic evaluation. European journal of cancer (Oxford, England : 1990). 2009;45(8):1397-406. doi:10.1016/j.ejca.2008.12.016.
6. Delea T, Amdahl J, Chit A, Amonkar M. Cost-effectiveness of lapatinib plus letrozole in her2-positive, hormone receptor–positive metastatic breast cancer in Canada. Current Oncology. 2013;20(5):e371.
7. Delea TE, Hawkes C, Amonkar MM, Lykopoulos K, Johnston SR. Cost-Effectiveness of Lapatinib plus Letrozole in Post-Menopausal Women with Hormone Receptor-and HER2-Positive Metastatic Breast Cancer. Breast care (Basel, Switzerland). 2013;8(6):429-37. doi:10.1159/000357316.
8. Delea TE, Tappenden P, Sofrygin O, Browning D, Amonkar MM, Karnon J et al. Cost-effectiveness of lapatinib plus capecitabine in women with HER2+ metastatic breast cancer who have received prior therapy with trastuzumab. The European journal of health economics : HEPAC : health economics in prevention and care. 2012;13(5):589-603. doi:10.1007/s10198-011-0323-1.
9. Elkin EB, Weinstein MC, Winer EP, Kuntz KM, Schnitt SJ, Weeks JC. HER-2 testing and trastuzumab therapy for metastatic breast cancer: a cost-effectiveness analysis. Journal of clinical oncology : official journal of the American Society of Clinical Oncology. 2004;22(5):854-63. doi:10.1200/jco.2004.04.158.
10. Frias C, Cortes J, Segui MA, Oyaguez I, Casado MA. Cost-effectiveness analyses of docetaxel versus paclitaxel once weekly in patients with metastatic breast cancer in progression following anthracycline chemotherapy, in Spain. Clinical & translational oncology : official publication of the Federation of Spanish Oncology Societies and of the National Cancer Institute of Mexico. 2010;12(10):692-700. doi:10.1007/s12094-010-0579-4.
11. Lazzaro C, Bordonaro R, Cognetti F, Fabi A, De Placido S, Arpino G et al. An Italian cost-effectiveness analysis of paclitaxel albumin (nab-paclitaxel) versus conventional paclitaxel for metastatic breast cancer patients: the COSTANza study. ClinicoEconomics and outcomes research: CEOR. 2013;5:125.
12. Le QA, Hay JW. Cost-effectiveness analysis of lapatinib in HER-2-positive advanced breast cancer. Cancer. 2009;115(3):489-98. doi:10.1002/cncr.24033.
13. Li N, van Agthoven M, Willemse P, Uyl-de Groot C. A cost--utility analysis comparing second-line chemotherapy schemes in patients with metastatic breast cancer. Anti-cancer drugs. 2001;12(6):533-40.

14. Lidgren M, Wilking N, Jonsson B, Rehnberg C. Cost-effectiveness of HER2 testing and trastuzumab therapy for metastatic breast cancer. Acta oncologica (Stockholm, Sweden). 2008;47(6):1018-28. doi:10.1080/02841860801901618.
15. Lopes G, Gluck S, Avancha K, Montero AJ. A cost effectiveness study of eribulin versus standard single-agent cytotoxic chemotherapy for women with previously treated metastatic breast cancer. Breast cancer research and treatment. 2013;137(1):187-93. doi:10.1007/s10549-012-2326-8.
16. Machado M, Einarson TR. Lapatinib in patients with metastatic breast cancer following initial treatment with trastuzumab: an economic analysis from the Brazilian public health care perspective. Breast Cancer: Targets and Therapy. 2012;4:173.
17. Matter-Walstra KW, Dedes KJ, Schwenkglenks M, Brauchli P, Szucs TD, Pestalozzi BC. Trastuzumab beyond progression: a cost-utility analysis. Annals of oncology : official journal of the European Society for Medical Oncology / ESMO. 2010;21(11):2161-8. doi:10.1093/annonc/mdq250.
18. Montero AJ, Avancha K, Gluck S, Lopes G. A cost-benefit analysis of bevacizumab in combination with paclitaxel in the first-line treatment of patients with metastatic breast cancer. Breast cancer research and treatment. 2012;132(2):747-51. doi:10.1007/s10549-011-1919-y.
19. Norum J, Risberg T, Olsen JA. A monoclonal antibody against HER-2 (trastuzumab) for metastatic breast cancer: a model-based cost-effectiveness analysis. Annals of oncology : official journal of the European Society for Medical Oncology / ESMO. 2005;16(6):909-14. doi:10.1093/annonc/mdi188.
20. Reed SD, Li Y, Anstrom KJ, Schulman KA. Cost effectiveness of ixabepilone plus capecitabine for metastatic breast cancer progressing after anthracycline and taxane treatment. Journal of clinical oncology : official journal of the American Society of Clinical Oncology. 2009;27(13):2185-91. doi:10.1200/jco.2008.19.6352.
21. Refaat T, Choi M, Gaber G, Kiel K, Mehta M, Gradishar W et al. Markov Model and Cost-Effectiveness Analysis of Bevacizumab in HER2-Negative Metastatic Breast Cancer. American journal of clinical oncology. 2014;37(5):480-5. doi:10.1097/COC.0b013e31827e4e9a.
22. Takeda AL, Jones J, Loveman E, Tan SC, Clegg AJ. The clinical effectiveness and cost-effectiveness of gemcitabine for metastatic breast cancer: a systematic review and economic evaluation. Health technology assessment (Winchester, England). 2007;11(19):iii, ix-xi, 1-62.
23. Verma S, Ilersich AL. Population-based pharmacoeconomic model for adopting capecitabine/docetaxel combination treatment for anthracycline-pretreated metastatic breast cancer. The oncologist. 2003;8(3):232-40.
24. Verma S, Maraninchi D, O'Shaughnessy J, Jamieson C, Jones S, Martin M et al. Capecitabine plus docetaxel combination therapy. Cancer. 2005;103(12):2455-65.
25. Miller K, Wang M, Gralow J, Dickler M, Cobleigh M, Perez EA et al. Paclitaxel plus bevacizumab versus paclitaxel alone for metastatic breast cancer. The New England journal of medicine. 2007;357(26):2666-76. doi:10.1056/NEJMoa072113.
